# Analysis of variation in calibration curves for Kodak XV radiographic film using model‐based parameters

**DOI:** 10.1120/jacmp.v11i4.3172

**Published:** 2010-08-05

**Authors:** Shu‐Hui Hsu, Ravi Kulasekere, Peter L. Roberson

**Affiliations:** ^1^ Department of Radiation Oncology University of Michigan, Ann Arbor Michigan 48109 U.S.A.

**Keywords:** radiographic film, calibration curve, film dosimetry, photon

## Abstract

Film calibration is time‐consuming work when dose accuracy is essential while working in a range of photon scatter environments. This study uses the single‐target single‐hit model of film response to fit the calibration curves as a function of calibration method, processor condition, field size and depth. Kodak XV film was irradiated perpendicular to the beam axis in a solid water phantom. Standard calibration films (one dose point per film) were irradiated at 90 cm source‐to‐surface distance (SSD) for various doses (16–128 cGy), depths (0.2, 0.5, 1.5, 5, 10 cm) and field sizes (5×5,10×10 and $20\times 20{\text{cm}}^{2}$). The 8‐field calibration method (eight dose points per film) was used as a reference for each experiment, taken at 95 cm SSD and 5 cm depth. The delivered doses were measured using an Attix parallel plate chamber for improved accuracy of dose estimation in the buildup region. Three fitting methods with one to three dose points per calibration curve were investigated for the field sizes of 5×5,10×10 and $20\times 20{\text{cm}}^{2}$. The inter‐day variation of model parameters (background, saturation and slope) were 1.8%, 5.7%, and 7.7% (1 σ) using the 8‐field method. The saturation parameter ratio of standard to 8‐field curves was 1.083±0.005. The slope parameter ratio of standard to 8‐field curves ranged from 0.99 to 1.05, depending on field size and depth. The slope parameter ratio decreases with increasing depth below 0.5 cm for the three field sizes. It increases with increasing depths above 0.5 cm. A calibration curve with one to three dose points fitted with the model is possible with 2% accuracy in film dosimetry for various irradiation conditions. The proposed fitting methods may reduce workload while providing energy dependence correction in radiographic film dosimetry. This study is limited to radiographic XV film with a Lumisys scanner.

PACS number: 87.55.Qr

## I. INTRODUCTION

Radiographic film dosimetry is an attractive method for various purposes in modern radiotherapy (e.g. the quality assurance of radiation beams and verification of dose distributions in phantoms). Advantages are convenience of use, good spatial resolution and two‐dimensional measurement. However, the challenges in radiographic film dosimetry include the dependence of response (optical density, OD) on: (1) photon beam energy, field size and depth in the phantom, (2) film orientation, (3) emulsion differences for batch, individual sheet, or sheet region, and (4) processing and densitometry conditions.^(^ ^1^ ^)^ Film response increases with increasing field size and depth beyond the depth of maximum dose. Palm et al.^(^ ^2^ ^)^ found that the film response strongly depends on the ratio of the number of photons below to the number of photons above 0.1 MeV (scatter‐to‐primary ratio). This scatter‐to‐primary ratio varies with field size and depth. It results in inaccurate dose determination when one calibration curve is used for all irradiation geometries. Several groups have studied the influence of energy dependence on film response.^(^ ^3^ ^–^ ^8^ ^)^ A large discrepancy in the magnitude of the variation of film response with field size and depth has been reported in these studies. Palm et al.^(^ ^9^ ^)^ reported that film response also depends on phantom material and extent, which contributed to the discrepancy in the magnitude of energy dependence reported in the literature. The influence of energy response could be reduced using Monte Carlo calculation to model the film response.^(^ ^2^ ^,^ ^10^ ^)^ Suchowerska et al.^(^ ^8^ ^)^ reported that film parallel orientation (to the beam axis) has an increased response compared to perpendicular orientation, up to 14% at 25 cm depth for a 6 MV photon beam. This could be attributed to the increased forward scattering of electrons in the silver halide for parallel orientation and the reduced attenuation of the beam when a gap appears between film and phantom.^(^ ^8^ ^,^ ^11^ ^)^ It is necessary to use the same orientations for calibration and experimental films. Bos et al.^(^ ^12^ ^)^ investigated interinstitutional variations of sensitometric curves. The OD variation at 50 cGy was up to 32%, and was primarily caused by film processing variations and, to a lesser degree, batch‐to‐batch variations. Therefore, a calibration curve (sensitometric curve) is usually acquired with each measurement to account for the influence of batch‐to‐batch and film processing variations.

Films must be carefully handled in order to achieve acceptable accuracy (e.g. 5%) for clinical verification purposes. The most challenging factors are the variation in film processing conditions and batch‐to‐batch sensitivity, which can be reduced by acquiring a calibration curve with 5–15 data points, trading off accuracy with workload.^(^ ^13^ ^)^ This workload increases the difficulty of reducing energy dependence of film response on the field size and depth, because it is difficult to acquire calibration curves for two or more irradiation conditions prior to the measurements. In this study, we used model‐based parameters to analyze the variation of calibration curves due to variations in the batch‐to‐batch and processing conditions, and energy dependence on field size and depth.

The calibration curve (OD versus dose) of XV film can be described by the single‐target single‐hit model:^(^ ^14^ ^,^ ^15^ ^)^

(1) $$OD=OD_{\max}(1‐e^{‐\alpha D})$$

where ${\text{OD}}_{\max}$ is the maximum optical density, α is a measure of film sensitivity, and *D* is the dose in cGy. The calibration curve can be determined if the parameters (${\text{OD}}_{\max}$ and α*)* are known for a specific batch, processing condition, energy, field size and depth. Due to the paucity of information on energy dependence in the buildup region, calibration curves were also acquired at superficial depths. In addition, we tested different methods to acquire the calibration curves with one to three data points. A few dose points can provide the possibility of acquiring calibration curves for two or more irradiation conditions while reducing the workload for film dosimetry.

## II. MATERIALS AND METHODS

### A. Phantom measurement and film handling

Kodak X‐Omat V Film (Eastman Kodak, Rochester, NY) with solid water phantom slabs (Gammex RMI Model 457, Middleton, WI) were used in this study. The lateral dimensions of the phantom slabs were 30×30 or $40\times 40\;{\text{cm}}^{2}$ with thicknesses from 0.2 cm to 5 cm. Film sheets were placed perpendicular to the beam axis. All measurements were performed with a 6 MV photon beam and 400 MU/min dose rate from a Varian 21EX accelerator (Varian, Palo Alto, CA) with a minimum of 10 cm backscatter depth.

All films were developed within a few hours after exposure using a Kodak X‐OMAT 3000RA Processor (Eastman Kodak, Rochester, NY), digitized with a Lumiscan75 laser scanner (Lumisys, Sunnyvale, CA), and analyzed using in‐house software. The film readout resolution was $0.123\times 0.123\;{\text{mm}}^{2}$. The pixel values for unexposed (background) and exposed films were extracted and averaged at the field centers using a region of interest (ROI) of $12.3\times 12.3\;{\text{mm}}^{2}$ and $1.23\times 1.23\;{\text{mm}}^{2}$, respectively.

In order to monitor the stability of the processor within a given specification, a standard sensitometric strip was exposed on the films during initial processing using a sensitometer (X‐Rite Incor., Grandville, MI) and processed together with experimental films. The film responses of three preselected levels were compared to established optical density reference levels (OD: 0.68, 1.18, 2.40) using a Nuclear Associates Model 07‐424 Digital Densitometer (Carle Place, NY). The processor temperature was maintained at 34.4°C±0.1°C.

### B. Single‐target single‐hit model

For a general theory of single‐target single‐hit model, see Zhu et al.^(^ ^15^ ^)^ In summary, the single‐target single‐hit model assumes that at least one event is necessary for the formation of a speck in the silver grain to achieve a probability of development.

(2) $$N/N_{0}=1‐e^{‐R}(\text{the}\;\text{proportion}\;\text{of}\;\text{developed}\;\text{grains})$$

where *N* is the number of developed grains per unit area, $N_{0}$ is the total number of grains per unit area in the emulsion, and *R* is the average number of events per grain. The average number of events (*R*) increases with increasing dose. In addition to the dose dependence of *R*, investigators have reported that a dose rate dependence exists for radiographic film (i.e. the Schwarzschild effect).^(^ ^16^ ^–^ ^18^ ^)^ The dose rate dependence is a minor effect compared to the dose dependence, so it could be ignored in our experiments.

The film processing conditions could affect the average number of events (*R*) and then change the number of developed grains. Therefore, *R* could be written as:

(3) $$R=\gamma \varepsilon \mu D_{w}$$

where $D_{w}=\text{dose to water}$, $\mu =\text{the energy dependence factor due to the photoelectric effect in film response}\;(\mu D_{w}:\text{dose to film})$, ε=film intrinsic sensitivity to the radiation dose, and γ=film processing effect. The optical density (OD) is used to describe the darkness of the film and is defined as:^(^ ^1^ ^)^

(4) $$OD=\log_{10}(I_{0}/I)$$

where $I_{0}$ is the incident light intensity measured in the absence of film and *I* is the intensity transmitted through the film. Then, the OD can be written as:

(5) $$OD=\log_{10}(e^{N\sigma })=(\log_{10}e)N_{0}\sigma (1‐e^{‐R})=OD_{\max}(1‐e^{‐\alpha D})$$

where σ is the effective area of a silver grain, ${\text{OD}}_{\max}$ is equal to $({\text{log}}_{10}e)N_{0}\sigma$ and would be constant for a constant number of grains in the emulation, and α is equal to γεμ and depends on the film processing conditions, film specific sensitivity and energy spectrum. Due to the limitation in the scanner (e.g. the nonlinearity for large OD and saturation at OD ~3.6 for the Lumisys scanner), it is difficult to acquire true ${\text{OD}}_{\max}$ through high‐dose beam delivery. A possible way of acquiring ${\text{OD}}_{\max}$ is through fitting the calibration curve with the model (Eq. 5).

The uniformity in the horizontal direction for the Lumiscan75 laser scanner is within 1% variation compared to the value at the center of the scanning region. The linearity of the scanner was evaluated with R‐squared of 0.997 for OD range between 0.2 and 3.0. Thus, the calibration curve (pixel value versus dose) in this study can be also described by the single‐target single‐hit model (Eq.1). The equation can be transformed as:^(^ ^13^ ^)^

(6) $$P=P_{0}+P_{s}(1‐e^{‐mD/P_{s}})$$

where *P* is the total pixel value, $P_{0}$ is the background pixel value, $P_{s}$ is the saturation pixel value, *m* is the film sensitivity slope parameter in pixel value/cGy, and *D* is the dose in cGy. The background pixel value ($P_{0}$) is due to film fog and base layer. If all silver grains were developed and the concentration of grains is assumed constant, $P_{s}$ can be assumed constant for each batch of film. The film sensitivity slope parameter (*m*) represents the initial slope of the response curve and depends on radiation type, energy, depth, field size, dose rate, film orientation and film processing conditions.^(^ ^1^ ^)^ The *m* parameter could be written as:

(7) $$m(E,FS,d,FP)=m_{E}(E,FS,d)\cdot m_{FP}(FP)$$

where *E*, *FS*, *d* and *FP* are the energy, field size, depth and film processing conditions, respectively. When the radiation type, machine output (MU/min) and film orientation are the same, $m_{E}(E,\;\text{FS},\;d)$ is a constant, independent of film processing conditions. The ratio of the *m* parameter in any irradiation condition to a reference condition can be determined at the same time (with less variation in film processing conditions) and the influence of $m_{\text{FP}}$ can be removed. Then, the *m* ratio for any irradiation condition is constant. The *m* parameter and whole calibration curve can be known for any irradiation condition when the reference calibration curve is acquired.

### C. Intra‐day and inter‐day variations of calibration curve using the 8‐field calibration method

**Figure 1:**
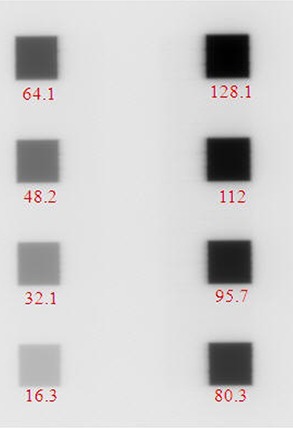
The intensity map of an 8‐field exposure on XV film. The numbers shown are dose in cGy for each subfield.

An 8‐field calibration method (eight dose points per film)^(^ ^19^ ^–^ ^21^ ^)^ was used to study the intra‐day and inter‐day variations in batch‐to‐batch and film processing conditions. The 8‐field calibration films were irradiated at 5 cm depth and 95 cm source‐to‐surface distance (SSD) with $3\times 3\;{\text{cm}}^{2}$ subfields (Fig. 1) and doses ranging from 16 to 128 cGy.^(^ ^20^ ^)^ The background was determined using an unexposed film from the same batch. The calibration curves for intra‐day variation were acquired on the same day (in 2006), while the calibration curves for inter‐day variation were collected over a 3‐year period (2004‐2007). Then, the calibration curves were fitted with Eq. (6), and both $P_{s}$ and *m* were obtained for each curve.

### D. Energy dependence in film dosimetry using the standard calibration method

A standard calibration method (one dose point per film) was used to study the energy dependence for various field sizes (5×5,10×10 and $20\times 20\;{\text{cm}}^{2}$, jaw only) and various depths (0.2, 0.5, 1.5, 5, 10 cm). Standard calibration films were irradiated at 90 cm source‐to‐surface distance (SSD) with a range of doses (16–128 cGy). The delivered doses were based on measurements using an Attix parallel plate chamber (RMI Model 449, Middleton, WI) in solid water for more accurate dose estimation in the buildup region. The number of data points acquired for each calibration curve was eight points for depths of 0.5–10 cm ($10\times 10{\text{cm}}^{2}$), three points for depths of 0.5–10 cm ($5\times 5\;{\text{cm}}^{2}$) and 0.2 cm ($10\times 10\;{\text{cm}}^{2}$), and one point for depths of 0.2–10 cm ($20\times 20\;{\text{cm}}^{2}$) and 0.2 cm ($5\times 5\;{\text{cm}}^{2}$). The background was determined using an unexposed film from the same batch. In addition, the 8‐field calibration curve was acquired on the same day for the comparison. These calibration curves were acquired on two different days (in 2006) and films were in two different batches.

The calibration curves for all field sizes and depths were fitted with Eq. (6). $P_{s}$ was assumed constant for the same batch of films. In order to get the optimal $P_{s}$ parameter to represent the saturated pixel value for each batch of films, two steps of fitting were performed. First, the data points after subtracting the background were fitted with Eq. (6) to get the $P_{s}$ parameters for each calibration curve. The optimal $P_{s}$ was acquired through averaging all $P_{s}$ values for films in the same batch, except the 8‐field calibration films. Second, the data points were refitted to get the *m* parameter with the optimal $P_{s}$ parameter for all standard curves, either for different field sizes or for different depths. The optimal $P_{s}$ acquired in the standard calibration method was compared with the $P_{s}$ acquired in the 8‐field calibration method. Because *m* does not only depend on the energy but also the processing condition, the ratio of *m* parameter in the standard method to that in the 8‐field method on the same day was calculated, in order to remove the influence from film processing conditions and to investigate energy dependence.

### E. The use of model‐based parameters to derive the calibration curve for a test dataset

As described in Section II. B., the *m* parameter depends on the energy, field size, depth and film processing conditions when the radiation type, machine output (MU/min) and film orientation are the same. Among these factors, the variation in film processing conditions is more significant. Methods to derive the calibration curves were investigated using model‐based parameters with one to three data points for different irradiation conditions. The methods to acquire the calibration curves include: (1) using the universal background ($P_{0}$) and saturated pixel values ($P_{s}$) with one and two exposed films as fitting dose points to acquire the *m* parameter (Method I), (2) using the universal saturated pixel value ($P_{s}$) and one background film with one and two fitting dose points to acquire $P_{0}$ and *m* parameter (Method II), and (3) one background film and two and three fitting dose points to acquire $P_{0}$, $P_{s}$ and *m* parameters (Method III). The derived calibration curves using the three methods were compared with the fitting curves using background film and eight fitting dose points. Three different irradiation geometries on different days were reported, including measurements: (1) at 10 cm depth, 90 cm SSD and $20\times 20\;{\text{cm}}^{2}$ field size, (2) at 5 cm depth, 95 cm SSD and $10\times 10\;{\text{cm}}^{2}$ field size, and (3) at 10 cm depth, 90 cm SSD and $5\times 5\;{\text{cm}}^{2}$ field size.

In addition, the *m* ratio for any irradiation condition should be constant, as described in Section II.B. The *m* parameter and whole calibration curve can be known for any irradiation condition when the reference calibration curve is acquired. In this study, the possibility of deriving standard calibration curves in different irradiation conditions through one reference calibration curve was investigated. The 8‐field calibration curve was used as a reference calibration curve. The parameter relationships (i.e., $P_{s}$ ratio and *m* ratio) between standard and 8‐field calibration curves were used to derive standard calibration curves at different depths (0.2, 0.5, 1.5, 10 cm) for a $20\times 20\;{\text{cm}}^{2}$ field size. These derived calibration curves were then compared to the true measured curves. The true curves were acquired on different days than the data used to determine the parameter relationships.

## III. RESULTS

### A. Intra‐day and inter‐day variations of calibration curve using the 8‐field calibration method

**Figure 2:**
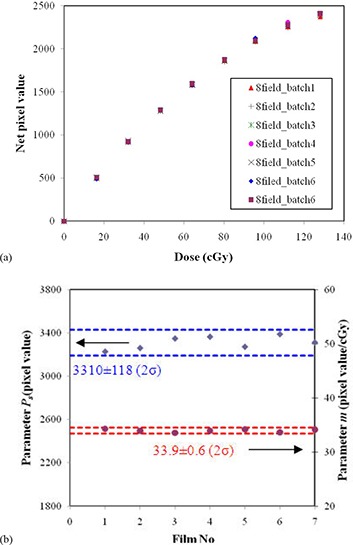
Intra‐day variations of calibration curves using the 8‐field method (a); intra‐day variations of parameters $P_{s}$ (diamond) and *m* (circle) for the 8‐field method (b) with the average and two standard deviations (2σ) shown. Film No. 6 and 7 were in the same batch (batch 6).

Figure 2(a) shows the 8‐field calibration curves (net pixel value vs. dose) acquired on the same day for different batches of films to study intra‐day variations. The last two films were in the same batch (batch 6). The curvatures are similar for all curves. Figure 2(b) shows the intra‐day variations of parameters $P_{s}$ and *m*. The averages of $P_{s}$ and *m* parameters were 3310 and 33.9(${\text{cGy}}^{-1}$) with standard deviations (1σ) of 1.8% and 0.8%, respectively. The variation of the $P_{s}$ value (1.8%) may represent variation of the concentration of grains on the film. The variation of parameter *m* (0.8%) for the 8‐field method is dominated by the variation of film processing conditions. The intra‐day variation of film processing conditions was observed to be smaller than batch‐to‐batch variation. The intra‐day variation due to the variation of processing conditions could be ignored.

**Figure 3:**
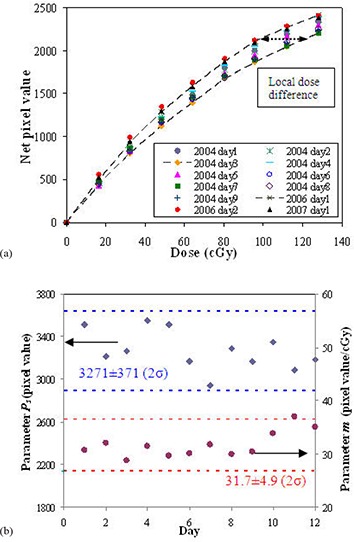
Inter‐day variation of calibration curves using the 8‐field method (a); inter‐day variations of parameters $P_{s}$ (diamond) and *m* (circle) for the 8‐field method (b) with the average and two standard deviations (2σ) shown.

**Figure 4:**
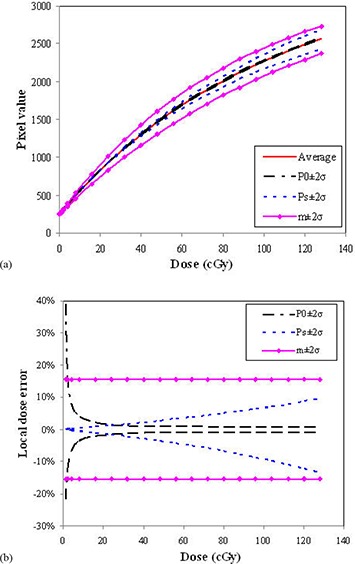
Model parameters influence on film response curves. The effects of parameters ($P_{0}$, $P_{s}$, *m*) at the 95% confidence level due to daily variation on the curvatures of calibration curves (a), and on the local dose error as a function of dose (b). Average calibration curve was calculated with $P_{0}$, $P_{s}$, and *m* parameters of 245, 3271 and 31.7, respectively.

Figure 3(a) shows the calibration curves using the 8‐field method obtained over three years (12 datasets). The curvature varies for different film batches and processing conditions. The inter‐day variation was up to 16% in local dose difference. Figure 3(b) shows the parameter variation ($P_{s}$ and *m*) in this period. The average of $P_{0}$, $P_{s}$ and *m* parameters were 245, 3271 and 31.7 ${\text{cGy}}^{-1}$ with standard deviations (1σ) of 1.8%, 5.7% and 7.7%, respectively. The inter‐day variation of film processing conditions was observed to be larger compared with batch‐to‐batch variation. Comparing intra‐day and inter‐day variations, the inter‐day variations are ~3 and ~10 times that of intra‐day variations in batch‐to‐batch and film processing conditions, respectively. Figure 4(a) shows the individual influences of variations of parameters ($P_{0}$, $P_{s}$, *m*) on the curvature of the film response. Figure 4(b) shows the effects of these parameters on the local dose errors in the dose range from 0 to 128 cGy when the average calibration curve (with $P_{0}$, $P_{s}$ and *m* parameters 245, 3271 and 31.7, respectively) is used. The dose error due to background variation is smaller than that due to film batch or processing condition variations. The background error leads to large errors in the low dose region (less than 10 cGy), but reduces to 1% or less at higher doses. The error in the $P_{s}$ parameter results in increasing local dose error with increasing dose. The error in the *m* parameter results in a constant relative error at all doses, and has the greatest influence on measurement error in the most useful film response range. Inter‐day dose error in the calibration curve is primarily due to a combination of film batch and processing condition variations.

### B. Energy dependence in film dosimetry using the standard calibration method

**Figure 5:**
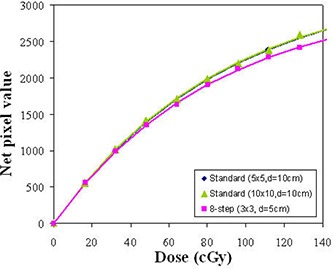
Calibration curves of 8‐field method at 95 cm SSD and standard methods for 5×5 and $10\times 10\;{\text{cm}}^{2}$ fields at 90 cm SSD.

Figure 5 shows comparisons of calibration curves using the 8‐field and standard methods on the same day. The difference in the curvature was found between two methods, but no significant difference was found between 5×5 and $10\times 10\;{\text{cm}}^{2}$ fields for the standard method. $P_{s}$ for the 8‐field method was consistently smaller than that for standard curves although the same batch of film was used. The ratio of $P_{s}$ parameter in the standard method to that in the 8‐field method was determined to be 1.083 with 0.5% in 1σ from measurements on the three different days. This difference was attributed to a characteristically different curvature to the 8‐field film response curve – probably a result of both the varying scatter conditions (particularly scatter cross‐talk between fields) and the nonuniformity in the horizontal direction of the scanner. This produces a systematic shift in the parameter values which are acquired from fitting the eight data points. Therefore, the $P_{s}$ value for the 8‐field method was not included in the search of optimal $P_{s}$ parameter to represent the saturation pixel value for the individual batch. While the $P_{s}$ parameter of 8‐field method cannot represent the standard $P_{s}$ value for a specific batch, its variation can represent the variation of the standard $P_{s}$ value.

**Figure 6:**
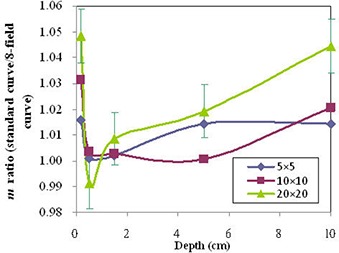
Sensitivity parameter (*m*) variations with radiation scatter conditions (field size and depth) as a ratio to the 8‐field method (fixed radiation geometry). The parameter ($P_{s}$) is constant for each batch of film. The error bar of ±1%(± 1σ) is shown for $20\times 20\;{\text{cm}}^{2}$ field. For other points, the estimated error is less than 1% because more data points were acquired for the calibration curve.

Figure 6 shows the characteristics of curvature as a function of field size and depth using the *m* ratio of standard to 8‐field methods. The estimated error of this ratio is ±1%. The ratio ranged from 0.99 to 1.05, depending on field size and depth. The ratio decreases with increasing depth at superficial depths shallower than 0.5 cm for the three field sizes. It increases with increasing depths deeper than 0.5 cm. Generally, the ratio increases with increasing field size. This result supports the dependence of the *m* ratio on the energy spectrum. The larger *m* ratio represents a larger film response for the same dose. At superficial depths, low energy scattered photons from the machine head have an influence on the film response, which results in an increased dose response, increasing with field size. At shallow depths, this influence decreases with increasing depth because of the decreasing fraction of head scattered photons. Beyond $d_{\max}$, the fraction of phantom scattered photons increases with depth and field size. Based on this figure, the systematic dose error would be ±3% if the calibration curve at 10 cm depth for the $10\times 10\;{\text{cm}}^{2}$ field is applied for the range of irradiation conditions shown in the figure. Our results are comparable to the study of Palm et al.^(^ ^9^ ^)^ In their study, the dose error was found to be within 5% for the same studied fields ($5\times 5,10\times 10,20\times 20\;{\text{cm}}^{2}$), depths ($d_{\max}$‐10 cm), and energy (6 MV photon beam) in a solid water phantom of 30 cm square.

### C. The use of model‐based parameters to derive the calibration curve for a test dataset

**Figure 7:**
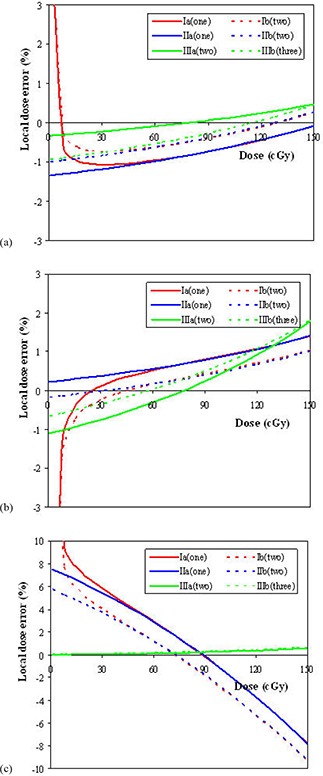
The local dose errors as a function of dose for geometry 1 ($20\times 20\;{\text{cm}}^{2}$) (a), geometry 2 ($10\times 10\;{\text{cm}}^{2}$) (b) and geometry 3 ($5\times 5\;{\text{cm}}^{2}$) (c) on the different days. The local dose error means the dose difference relative to the true dose for the same pixel value.

Figure 7 shows the local dose error when universal parameters are used. Table 1 shows the individual parameters for different methods and different irradiation geometries. From the result for 8‐field method in Section III. A, the universal $P_{0}$ was 245, and the universal $P_{s}$ was 3542 after corrected with the ratio of standard to 8‐field methods (1.083). For geometry 1 and 2, the calibration curves using different fitting methods are close, with the local dose error within 2% for the dose range from 10 to 150 cGy. The local dose error is large for the dose below 10 cGy for Method I. This is because the universal background value was used (Table 1). However, the influence can be ignored because the absolute dose difference is within 0.1 cGy. For geometry 3, Methods I and II lead to large errors, compared to Method III. This is because the true $P_{s}$ is 9% less than the universal $P_{s}$. Therefore, the universal $P_{s}$ cannot be applied in the situation where the saturated pixel value is significantly different from the universal $P_{s}$ (e.g., 5% difference), because it would lead to unacceptable local dose error (> 5%). In the situation of the larger variation in $P_{s}$, Method III is preferable, because the $P_{s}$ is determined from data points. The local dose error is reduced to be within 1%. However, at least two data points are needed for Method III. Comparing one and two data points in geometry 1 and geometry 2, no significant reduction in local dose error was found when two data points were used in the data fitting.

**Table 1:** The individual parameters for different fitting methods and different irradiation geometries.

*Geometry*	*1*	*2*	*3*
SSD (cm)	90	95	90
Field size (${\text{cm}}^{2}$)	20×20	10×10	5×5
Depth (cm)	10	5	10
*Fitting parameters with one background and eight dose points*			
$P_{0}$	247	240	254
$P_{s}$	3585	3577	3209
*m*	30.80	33.58	40.18
*Method I: Universal* $P_{s}$ and $P_{0}$			
Universal $P_{0}$	245	245	245
Universal $P_{s}$	3542	3542	3542
Ia (one point^a^): *m*	31.28	33.37	37.62
Ib (two points^b^): *m*	31.17	33.48	38.28
*Method II: Universal* $P_{s}$			
$P_{0}$	247	240	254
Universal $P_{s}$	3542	3542	3542
IIa (one point^a^): *m*	31.23	33.50	37.36
IIb (two points^b^): *m*	31.11	33.63	37.98
*Method III*			
$P_{0}$	247	240	254
IIIa (two points^c^): $P_{s}$	3558	3494	3200
IIIb (three points^d^): $P_{s}$	3538	3506	3200
IIIa (two points^c^): *m*	30.91	33.95	40.19
IIIb (three points^d^): *m*	31.10	33.80	40.16

a Dose point: ~80 cGy

b Dose points: ~40 and ~80 cGy

c Dose points: ~40 and ~120 cGy

d Dose points: ~40, ~80 and ~120 cGy

For the derived (calculated) standard calibration curves using the 8‐field exposure compared with depth‐specific measured standard calibration curves, the average local dose differences were −1%, 3.5%, 0.4%, and 2.5% at 0.2, 0.5, 1.5, and 10 cm depths, respectively.

### D. Uncertainty analysis

From Eq. (6), the dose can be expressed as:

(8) $$D=‐\frac{P_{s}}{m}\ln(1‐\frac{P‐P_{0}}{P_{s}})$$

Based on error propagation, the dose error arises from systematic errors in the determination of $P_{s}$ and *m* parameters and random errors in the measurement data. Using propagation of errors, estimated at midrange of film response $\left(\frac{P‐P_{0}}{P_{s}}\sim 0.5\right)$,

(9) $$\sigma_{D}^{2}=[{\left(\frac{\partial D}{\partial P_{s}}\right)}^{2}\sigma_{P_{s}}^{2}+{\left(\frac{\partial D}{\partial m}\right)}^{2}\sigma_{m}^{2}]+[{\left(\frac{\partial D}{\partial P}\right)}^{2}\sigma_{P}^{2}+{\left(\frac{\partial D}{\partial P_{0}}\right)}^{2}\sigma_{P_{0}}^{2}]$$

Equation (9) can be transformed to:

(10) $${\left(\frac{\sigma_{D}}{D}\right)}^{2}=[0.25(\frac{\sigma_{P_{s}}}{P_{s}})+{\left(\frac{\sigma_{m}}{m}\right)}^{2}]+[3{\left(\frac{\sigma_{P}}{P}\right)}^{2}+0.05{\left(\frac{\sigma_{P_{0}}}{P_{0}}\right)}^{2}]$$

The first bracket represents the systematic error component from repeated measurements and the second bracket represents the random error component. Using estimated values for the σ's (0.5%, 1%, 1%, 0.5% for $\frac{\sigma_{P_{s}}}{P_{s}}$, $\frac{\sigma_{m}}{m}$, $\frac{\sigma_{P}}{P}$, $\frac{\sigma_{P_{0}}}{P_{0}}$ respectively):

(11) $$\frac{\sigma_{D}}{D}\sim 2\%$$

The estimated error is 2% when the model‐based parameters are used to derive the calibration curves. Results in Section III.C for depth‐specific curves were consistent with this estimated uncertainty − ~4% within 95% confidence level. Compared to the systematic error of 3% in energy dependence with estimated random error of 1.5% (1σ), the total uncertainty (2σ) would be up to 6% within 95% confidence level.

## IV. DISCUSSION

From the results, the error due to the variation in film processing conditions is more significant than the error due to the batch‐to‐batch variation and energy dependence. It is necessary to acquire a calibration curve with enough dose points prior to the measurements to reflect the inter‐day variation of film processing conditions. Therefore, the universal $m_{\text{FP}}$ in Eq. (7) cannot be determined unless the film processing conditions are well known. Although the universal $m_{\text{FP}}$ is not possible, the universal $m_{E}$ in Eq. (7) could be possible. The $m_{E}$ parameter cannot be known for any irradiation condition when $m_{\text{FP}}$ is unknown. However, the $m_{E}$ relationship could be calculated and is constant when $m_{\text{FP}}$ is controlled (i.e. stable film processing conditions). Once the $m_{E}$ relationship between any irradiation condition and a reference condition is known, the *m* parameter for other irradiation conditions can be calculated using the *m* parameter for the reference condition. Therefore, calibration curves for other irradiation conditions could be derived using the reference curve. The preliminary investigation of this possibility shows that the accuracy could be within 4% (95% confidence level), including the systematic and random errors. Based on Fig. 6, if the calibration curve at 10 cm depth for $10\times 10\;{\text{cm}}^{2}$ field is applied for the field sizes from 5×5 to $20\times 20\;{\text{cm}}^{2}$ and depth from 0.2 cm to 10 cm, the systematic error would be within 3% due to the energy dependence. Under this condition, calibration curves specific to field size and depth show a modest improvement (~ 2%). However, when the measurement is done for the field size larger than $20\times 20\;{\text{cm}}^{2}$, at depth deeper than 10 cm or with different phantom materials (e.g. acrylic and polystyrene),^(^ ^9^ ^)^ calibration curves specific to these irradiation conditions would show more improvement. Under this condition, the use of the universal $m_{E}$ relationship would be helpful to improve the accuracy in film dosimetry for reasonable effort.

A universal background value is possible because the random variation is smaller than 2% (1σ) and it primarily affects the dose below 10 cGy. A universal saturated pixel value is possible only when the batch‐to‐batch difference is smaller than 5% so that the dose error could be within 5%. When the batch‐to‐batch difference is larger than 5%, a significant dose error would appear using a universal saturated pixel value. In this situation, using more data points to acquire all three parameters is preferable. However, the dose points for fitting must be carefully selected because the selected dose points would significantly affect the accuracy of the calibration curves. One dose point close to 120 cGy is recommended in order to get a more accurate $P_{s}$. Conclusively, a calibration curve with one to three dose points fitted with single‐target single‐hit model is possible with 2% accuracy in film dosimetry for various irradiation conditions.

In routine clinical use, one can acquire a calibration curve with two to three dose points fitted with the single‐target single‐hit model when the saturated pixel value is unknown for a certain batch. Once the saturated pixel value is known for the batch, one can use one to two dose points fitted with the model and constant saturated pixel value. The use of a constant background and a saturated pixel value for acquiring a calibration curve would depend on measurement goals. However, it is not necessary to use many dose points for acquiring a calibration curve with comparable accuracy. In addition, if calibration curves for various irradiation conditions are acquired at the same time, one can use this information to establish the energy‐dependent parameter ($m_{E}$) relationship for future use.

This study is limited to radiographic XV film with a Lumisys scanner. For other types of densitometers, the characteristics must be determined before the proposed fitting method can be used. For other types of radiographic film, an appropriate response model is required.^(^ ^15^ ^)^

## V. CONCLUSIONS

Film dosimetry can provide valuable information in radiotherapy. However, the workload increases with increasing accuracy due to its response dependence on energy, batch and film processing conditions. This study has used model‐based parameters to analyze the intra‐day and inter‐day variations of calibration curves due to batch‐to‐batch, film processing and energy dependences. In addition, the possibility of acquiring calibration curves with one to three dose points was investigated. The background error (fog and base layer) leads to large error in the low dose region (less than 10 cGy). The error in the saturation pixel value (grain concentration) results in increasing local dose error with increasing dose, but does not become dominant until near saturation dose. The error in the film sensitivity parameter (due to film processing conditions, field size and depth) results in a constant error for the useful dose measurement range, and has the most significant influence on film response curves. Acquiring a calibration curve with one to three dose points to correct the film processing variation is possible with an accuracy of 2%. This method may reduce the workload in film dosimetry while acquiring calibration curves specific to the irradiation conditions. This study is limited to radiographic XV film with a Lumisys scanner.

## References

[1] Pai S , Das IJ , Dempsey JF , et al. TG‐69: radiographic film for megavoltage beam dosimetry. Med Phys. 2007;34(6):2228–58. 17654924 10.1118/1.2736779

[2] Palm A , Kirov AS , LoSasso T . Predicting energy response of radiographic film in a 6 MV x‐ray beam using Monte Carlo calculated fluence spectra and absorbed dose. Med Phys. 2004;31(12):3168–78. 15651599 10.1118/1.1812911

[3] Danciu C , Proimos BS , Rosenwald JC , Mijnheer BJ . Variation of sensitometric curves of radiographic films in high energy photon beams. Med Phys. 2001;28(6):966–74. 11439493 10.1118/1.1376443

[4] van Battum LJ , Heijmen BJ . Film dosimetry in water in a 23 MV therapeutic photon beam. Radiother Oncol. 1995;34(2):152–59. 7597214 10.1016/0167-8140(94)01500-3

[5] van Bree NA , Idzes MH , Huizenga H , Mijnheer BJ . Film dosimetry for radiotherapy treatment planning verification of a 6 MV tangential breast irradiation. Radiother Oncol. 1994;31(3):251–55. 8066209 10.1016/0167-8140(94)90431-6

[6] Sykes JR , James HV , Williams PC . How much does film sensitivity increase at depth for larger field sizes? Med Phys. 1999;26(2):329–30. 10076992 10.1118/1.598520

[7] Burch SE , Kearfott KJ , Trueblood JH , Sheils WC , Yeo JI , Wang CK . A new approach to film dosimetry for high energy photon beams: lateral scatter filtering. Med Phys. 1997;24(5):775–83. 9167171 10.1118/1.597999

[8] Suchowerska N , Hoban P , Davison A , Metcalfe P . Perturbation of radiotherapy beams by radiographic film: measurements and Monte Carlo simulations. Phys Med Biol. 1999;44(7):1755–65. 10442711 10.1088/0031-9155/44/7/314

[9] Palm A , LoSasso T . Influence of phantom material and phantom size on radiographic film response in therapy photon beams. Med Phys. 2005;32(8):2434–42. 16193772 10.1118/1.1949747

[10] Kirov AS , Caravelli G , Palm A , Chui C , LoSasso T . Pencil beam approach for correcting the energy dependence artifact in film dosimetry for IMRT verification. Med Phys. 2006;33(10):3690–99. 17089835 10.1118/1.2229425

[11] Suchowerska N , Hoban P , Butson M , Davison A , Metcalfe P . Directional dependence in film dosimetry: radiographic and radiochromic film. Phys Med Biol. 2001;46(5):1391–97. 11384060 10.1088/0031-9155/46/5/305

[12] Bos LJ , Danciu C , Cheng CW , et al. Interinstitutional variations of sensitometric curves of radiographic dosimetric films. Med Phys. 2002;29(8):1772–80. 12201424 10.1118/1.1494833

[13] van Battum LJ , Huizenga H . The curvature of sensitometric curves for Kodak XV‐2 film irradiated with photon and electron beams. Med Phys. 2006;33(7):2396–403. 16898442 10.1118/1.2207130

[14] Williamson JF , Khan FM , Sharma SC . Film dosimetry of megavoltage photon beams: a practical method of isodensity‐to‐isodose curve conversion. Med Phys. 1981;8(1):94–98. 7207433 10.1118/1.594913

[15] Zhu XR , Yoo S , Jursinic PA , et al. Characteristics of sensitometric curves of radiographic films. Med Phys. 2003;30(5):912–19. 12773000 10.1118/1.1568979

[16] Djouguela A , Kollhoff R , Rubach A , Harder D , Poppe B . The Schwarzschild effect of the dosimetry film Kodak EDR 2. Phys Med Biol. 2005;50(21):N317–21. 16237231 10.1088/0031-9155/50/21/N04

[17] Djouguela A , Kollhoff R , Rühmann A , Willborn KC , Harder D , Poppe B . Physical mechanism of the Schwarzschild effect in film dosimetry – theoretical model and comparison with experiments. Phys Med Biol. 2006;51(17):4345–56. 16912385 10.1088/0031-9155/51/17/014

[18] Martens C , Claeys I , De Wagter C , De Neve W . The value of radiographic film for the characterization of intensity‐modulated beams. Phys Med Biol. 2002;47(13):2221–34. 12164583 10.1088/0031-9155/47/13/303

[19] Childress NL , Dong L , Rosen II . Rapid radiographic film calibration for IMRT verification using automated MLC fields. Med Phys. 2002;29(10):2384–90. 12408313 10.1118/1.1509441

[20] Kulasekere R , Moran JM , Fraass BA , Roberson PL . Accuracy of rapid radiographic film calibration for intensity‐modulated radiation therapy verification. J Appl Clin Med Phys. 2006;7(2):86–95. 10.1120/jacmp.v7i2.2202 PMC5722446 17533325

[21] Ritt DM , inventor. Automated calibration for radiation dosimetry using fixed or moving beams and detectors. United States patent US 6675116. 2004 Jan 6.

